# Aberrant cGMP signaling persists during recovery in mice with oxygen-induced pulmonary hypertension

**DOI:** 10.1371/journal.pone.0180957

**Published:** 2017-08-09

**Authors:** Marta Perez, Keng Jin Lee, Herminio J. Cardona, Joann M. Taylor, Mary E. Robbins, Gregory B. Waypa, Sara K. Berkelhamer, Kathryn N. Farrow

**Affiliations:** 1 Department of Pediatrics, Northwestern University, Chicago, IL, United States of America; 2 Department of Pediatrics, University at Buffalo, Buffalo, NY, United States of America; University of Illinois at Chicago College of Medicine, UNITED STATES

## Abstract

Bronchopulmonary dysplasia (BPD), a common complication of preterm birth, is associated with pulmonary hypertension (PH) in 25% of infants with moderate to severe BPD. Neonatal mice exposed to hyperoxia for 14d develop lung disease similar to BPD, with evidence of associated PH. The cyclic guanosine monophosphate (cGMP) signaling pathway has not been well studied in BPD-associated PH. In addition, there is little data about the natural history of hyperoxia-induced PH in mice or the utility of phosphodiesterase-5 (PDE5) inhibition in established disease. C57BL/6 mice were placed in room air or 75% O_2_ within 24h of birth for 14d, followed by recovery in room air for an additional 7 days (21d). Additional pups were treated with either vehicle or sildenafil for 7d during room air recovery. Mean alveolar area, pulmonary artery (PA) medial wall thickness (MWT), RVH, and vessel density were evaluated at 21d. PA protein from 21d animals was analyzed for soluble guanylate cyclase (sGC) activity, PDE5 activity, and cGMP levels. Neonatal hyperoxia exposure results in persistent alveolar simplification, RVH, decreased vessel density, increased MWT, and disrupted cGMP signaling despite a period of room air recovery. Delayed treatment with sildenafil during room air recovery is associated with improved RVH and decreased PA PDE5 activity, but does not have significant effects on alveolar simplification, PA remodeling, or vessel density. These data are consistent with clinical studies suggesting inconsistent effects of sildenafil treatment in infants with BPD-associated PH.

## Introduction

Bronchopulmonary dysplasia (BPD) remains a significant contributor to late morbidity and mortality of formerly premature infants [1]. A subset of infants with BPD will go on to develop pulmonary hypertension (PH), which can result in right ventricular hypertrophy (RVH) and right ventricular failure. Not surprisingly, development of PH is associated with longer length of hospitalization and increased healthcare costs [2–5]. In addition, recent reports indicate that mortality from BPD-associated PH is as high as 50% by 2 years [3]. Despite significant advances in neonatal critical care, the best treatment options for BPD infants with PH have not been defined. Furthermore, the underlying mechanisms and signaling pathways involved in the development of BPD-associated PH remain poorly understood.

In the current study, we utilized a murine model of hyperoxic lung injury to mimic BPD. Work from our group as well as others demonstrates that chronic hyperoxia exposure of neonatal mice leads to alveolar simplification and features of PH, including development of RVH and remodeling of small pulmonary arteries (PAs) [6–8]. It is now becoming increasingly evident that even brief exposures of neonatal rodents to hyperoxia can result in a similar phenotype. Exposure of newborn mice to 4 days of 100% O_2_ results in a shortened life span, changes in lung function, and cardiovascular alterations [9]. Our lab has previously demonstrated that oxygen exposures as short as 24h result in RVH that persists at 14d of age, and exposures of 72h result in changes in alveolar area, radial alveolar count, septal length, RVH, and Nox1 signaling that persist at 14d of age [6, 10]. Significant questions remain, however, about long-term consequences of neonatal hyperoxia exposure as well as the specific signaling pathways involved.

We have previously shown that alterations in the nitric oxide-cyclic guanosine monophosphate (NO-cGMP) signaling pathway, a major regulator of pulmonary vascular tone, are important contributors to the pathophysiology of BPD-associated PH [6]. Two key enzymes and potential therapeutic targets in this pathway include phosphodiesterase-5 (PDE5) and soluble guanylate cyclase (sGC). PDE5 contributes to vasoconstriction through degradation of cGMP, while sGC is important in maintenance of vasodilation by increasing cGMP levels. Sildenafil, a PDE5 inhibitor, at high doses has been shown to improve alveolar growth and decrease RVH in rodent models of hyperoxic-lung injury [11–13] and in an adult rodent monocrotaline-induced PH model [14, 15]. We have previously demonstrated that low-dose sildenafil given concurrent with a two-week hyperoxia exposure attenuates markers of PH, normalizes PA PDE5 activity, and increases cGMP levels in the mouse model of hyperoxic lung injury with PH [6].

In the current study, we set out to determine whether neonatal oxygen exposure results in persistent cardiopulmonary abnormalities and aberrant NO-cGMP signaling during room air recovery and to determine the effects of rescue treatment with sildenafil, since this is the most common clinical scenario encountered [16–19]. We found that exposure of neonatal mice to hyperoxia during the first 2 weeks of life results in alveolar simplification, RVH, decreased vessel density, and increased medial wall thickness (MWT), as well as abnormalities in NO-cGMP signaling, including increased PDE5 activity, that persist for 1 week after returning to room air. Sildenafil treatment during the first week of recovery was associated with a more rapid improvement in RVH but did not impact alveolar simplification, remodeling of small PAs, or vessel density. In addition, sildenafil treatment was associated with decreased PA PDE5 activity.

## Materials and methods

### Animal protocols

This study was approved by the Institutional Animal Care and Use Committee at Northwestern University. Mice were housed at 22±2°C on a 12h day/night cycle and fed a standard diet of rodent chow and water ad libitum. Aged-matched C57BL/6 litters (Charles River, Wilmington, MA) were placed in room air (control) or 75% O_2_ (Chronic Hyperoxia, CH) in a Plexiglas chamber (Biospherix, Lacona, NY) within the first 24h of birth [6]. Dams were rotated every 24h to prevent oxygen toxicity to the adult animals. After 14d of exposure, pups were allowed to recover at room air for an additional 7 days (21d) before euthanasia. Mice were observed at least once a day. According to our established protocol mice would be removed from the study and euthanized if showing signs of distress including lethargy, respiratory distress, color, and behavior changes. No mice died or had to be euthanized in these studies prior to the experimental endpoint. Euthanasia was performed by a lethal overdose of isoflurane followed by bilateral thoracotomy and tissue harvest.

### Sildenafil treatment during recovery

Litters of 21d control and CH pups received sildenafil (Revatio [0.8 mg/ml sildenafil in 5% dextrose], Pfizer, New York, NY) or vehicle (5% dextrose) injections (3 mg/kg/dose, subcutaneous) every other day starting from P14, totaling four doses.

### Measurement of right ventricular hypertrophy

Hearts were harvested and dissected to separate the right ventricle (RV) and left ventricle plus septum (LV+S). Fulton’s Index (RV weight divided by LV+S weight) was used to assess RVH [6].

### Measurement of medial wall thickness

MWT of small PAs was assessed as a measure of pulmonary hypertension. Mouse lungs were inflation fixed at 25 cm H_2_O using 4% formalin, stained with hematoxylin and eosin (H&E), and imaged using an Olympus BX40 microscope (Olympus America, Melville, NY) at 40X with Pixera software (Pixera Corporation, Santa Clara, CA). 8–10 images per animal were taken and analyzed in a blinded fashion. MWT was measured as the ratio of the area of small PA (<100 μM) wall over the total PA area as previously described [6].

### Measurement of alveolar area and chord length

Lung sections were stained with hematoxylin for 16h, and lung morphometry images were taken using an Olympus BX40 microscope (Olympus America, Melville, NY) at 20X with Pixera software (Pixera Corporation, Santa Clara, CA) as previously described [6, 20]. 8–10 images per animal were taken and analyzed in a blinded fashion. Mean alveolar area and chord length were measured using Scion software (Scion Corporation, Frederick, MD).

### Measurement of alveolar counts and septal thickness

Lung sections were stained with H&E and images were taken using an Olympus BX40 microscope (Olympus America, Melville, NY) at 20X with Image Pro Plus 4.0 software (Media Cybernetics, Silver Spring, MD). 4 images per animal were taken and analyzed in a blinded fashion. Alveolar counts and septal thickness were measured using research-based digital image analysis software (Image Pro Plus 4.0; Media Cybernetics, Silver Spring, MD) [21].

### Immunohistochemistry

5 μm thick lung sections were rehydrated then treated with Proteinase K (Sigma-Aldrich, St. Louis, MO, 20 μg/ml) in TE buffer (50 mM Tris Base, Amresco, Solon, OH, 1 mM EDTA, Fisher Scientific, Waltham, MA, 0.5% Triton X-100, Fisher Scientific, pH 8) in a humidified chamber at 37°C. After 15 minutes, sections were allowed to return to room temperature for 10 minutes and rinsed with Tris-buffered saline containing 0.1% Tween-20 (TBST) before being blocked at room temperature with 5% bovine serum albumin (BSA) in TBST for 30 minutes. Sections were incubated with von Willebrand Factor (vWF) primary antibody (Dako, Carpenteria, CA; 1:50 dilution overnight at 4°C), followed by Rhodamine Red-anti-rabbit secondary antibody (Fisher Scientific, 1:100 dilution, 1 hour at room temperature) in blocking solution as previously described [6, 10].

8 images per animal were randomly captured with a Nikon Eclipse TE-300 fluorescent microscope under 10X magnification. Small vessels (<100 μM) were counted and averaged per animal in a blinded fashion.

### Pulmonary artery protein

Mouse lungs were flushed with phosphate buffered saline (PBS, Mediatech, Manassas, VA) through the right ventricle followed by a solution of small iron particles (0.2 μm) that lodge into the small PAs. The lungs were then inflated with 1% w/v of agarose (Sigma-Aldrich), chilled in cold PBS and minced. Minced lungs were treated with collagenase (Sigma-Aldrich, 80 U/ml) for 1 hour followed by manual dissociation. Iron-filled vessels were lysed in 1X Mg-lysis buffer (Upstate, Charlottesville, VA) supplemented with a protease inhibitor cocktail (Sigma-Aldrich) and a phosphatase inhibitor cocktail (EMD Biosciences, San Diego, CA) and sonicated. Released iron particles were removed using a Dynal Magnetic Particle Concentrator (Invitrogen, Grand Island, NY) [22]. PA lysates were analyzed for PDE5 and soluble guanylate cyclase (sGC) activity as described below.

### Western blot analysis

PA protein expression was assessed via Western blot for sGCα, sGCβ and PDE5 as previously described [6, 23]. Briefly, membranes were blocked for 1 hour at room temperature with 5% BSA + 1X TBST then incubated overnight at 4°C with primary antibody in 5% BSA + 1X TBST at an appropriate dilution (1:2000 for rabbit anti-sGCα [Caymen Chemical, Ann Arbor, MI], 1:2000 for rabbit anti-sGCβ [Caymen Chemical], and 1:2000 for rabbit anti-PDE5 [Santa Cruz Biotechnology, Santa Cruz, CA]) or for 1 hour at room temperature with primary antibody for β-actin (1:5000 for mouse anti-β-actin [Sigma-Aldrich]) in 5% BSA +1X TBST. The membranes were washed and incubated with the appropriate secondary antibody diluted 1:2000 in 5% BSA + 1X TBST for sGCα, sGCβ and PDE5 (Cell Signaling) or 1:5000 for β-actin (Cell Signaling). Membranes were then washed and exposed via chemiluminescence (Thermo Fischer Scientific, Rockford, IL). Bands were analyzed using Image Lab (BioRad, Hercules, CA). Expression was normalized to β-actin. Data are shown as fold relative to corresponding controls.

### PDE5 activity assay

PA protein was first purified using a Centri-Spin 10 column to remove all free phosphates (Princeton Separations, Adelphia, NJ). Samples were analyzed in duplicate for cGMP-hydrolytic activity using a commercially available colorimetric cyclic nucleotide phosphodiesterase assay kit (Enzo, Farmingdale, NY) with and without 100 nM of sildenafil (Sigma-Aldrich) as previously described [6, 23]. Protein concentration was determined using the Bradford method [22]. Results are shown as the PDE5-specific pmol cGMP hydrolyzed/minute/mg of total purified protein.

### sGC activity assay

30 μg of PA protein was measured using the Bradford method. Samples were incubated in a reaction mixture of 50 mM Tris-HCl (pH 7.5, Fisher Scientific), 4 mM MgCl_2_ (Fisher Scientific), 0.5 mM 3-isobutyl-1-methylxanthine (Enzo), 7.5 mM creatine phosphate (Sigma-Aldrich), 0.2 mg/mL creatine phosphokinase (Sigma-Aldrich), 1 mM sodium nitroprusside (Sigma-Aldrich), and 1 mM GTP (Sigma-Aldrich) at 37°C. After 10 minutes, the reaction was terminated using HCl (Sigma-Aldrich) to a final concentration of 0.1 N. Samples were dried using a Speed-Vac, and pellets were resuspended in 100–200 μL of cGMP EIA buffer (Cayman Chemical, Ann Arbor, MI). cGMP levels in the samples were measured in duplicate using a commercially available EIA kit (Cayman Chemical). Results were measured using a Bio-Rad iMark automated plate reader (Hercules, CA) at 405 nm [6, 23]. sGC activity results are shown as pmol cGMP/minute/mg total protein.

### Cyclic GMP enzyme immunoassay (EIA)

Briefly, small PAs from 21d mice were isolated as described in the PA protein isolation. After manual dissociation the iron-filled vessels were lysed with 0.1 N HCl to inactivate the endogenous phosphodiesterases. Samples were then purified as per manufacturer’s protocol (Cayman Chemical). cGMP levels were measured by EIA in duplicate, using a commercially available kit as previously described [6]. Results were measured using a Bio-Rad iMark automated plate reader at 405 nm. Results are shown as pmol cGMP/mL/mg total protein.

### Statistical analysis

Data are expressed as dot plots with each dot representing an individual animal with means and SEM represented by horizontal lines. Results were analyzed by unpaired t-test or ANOVA with post-hoc Bonferroni’s analysis using Prism software version 7.0 (Graphpad Software Inc., San Diego, CA) with significance set at p≤0.05.

## Results

### Neonatal mice demonstrate alveolar simplification, RVH and decreased vessel density after 14d of chronic oxygen exposure

**Fig 1:**
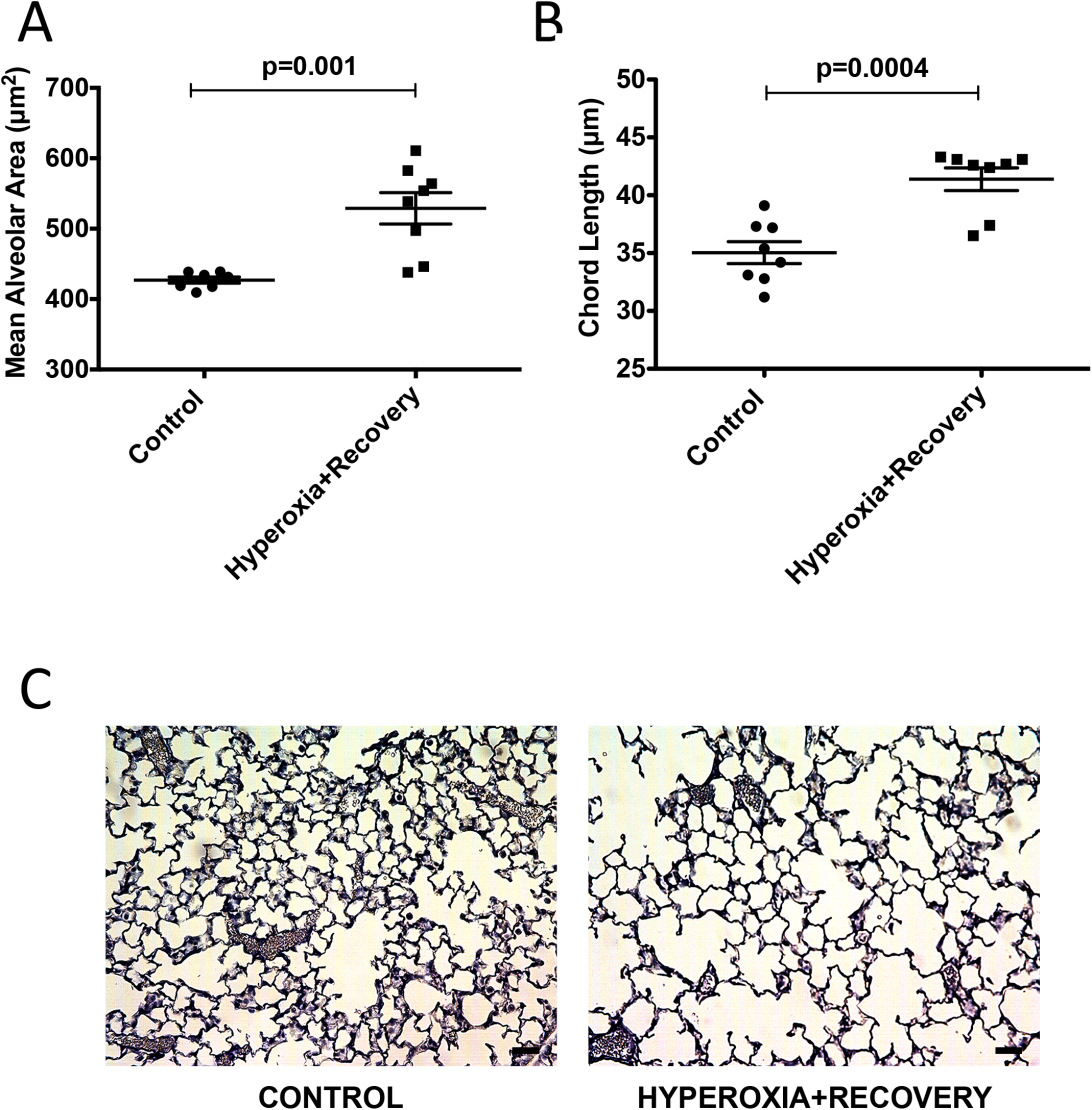
Hyperoxia exposure of neonatal mice is associated with persistent alveolar simplification. Mice were exposed to hyperoxia or room air for 14d, followed by recovery in room air for an additional 7d (21d). (A) Mean alveolar area and (n = 7 animals per group). (B) chord length were measured using Scion software (n = 8 animals per group). (C) Representative hematoxylin-stained lung sections are shown for control 21d and hyperoxia + recovery 21d. Bar = 50 μm. Data are expressed as dot plots with each dot representing individual animals with means and SEM represented by horizontal lines. Results were analyzed using an unpaired t-test.

**Fig 2:**
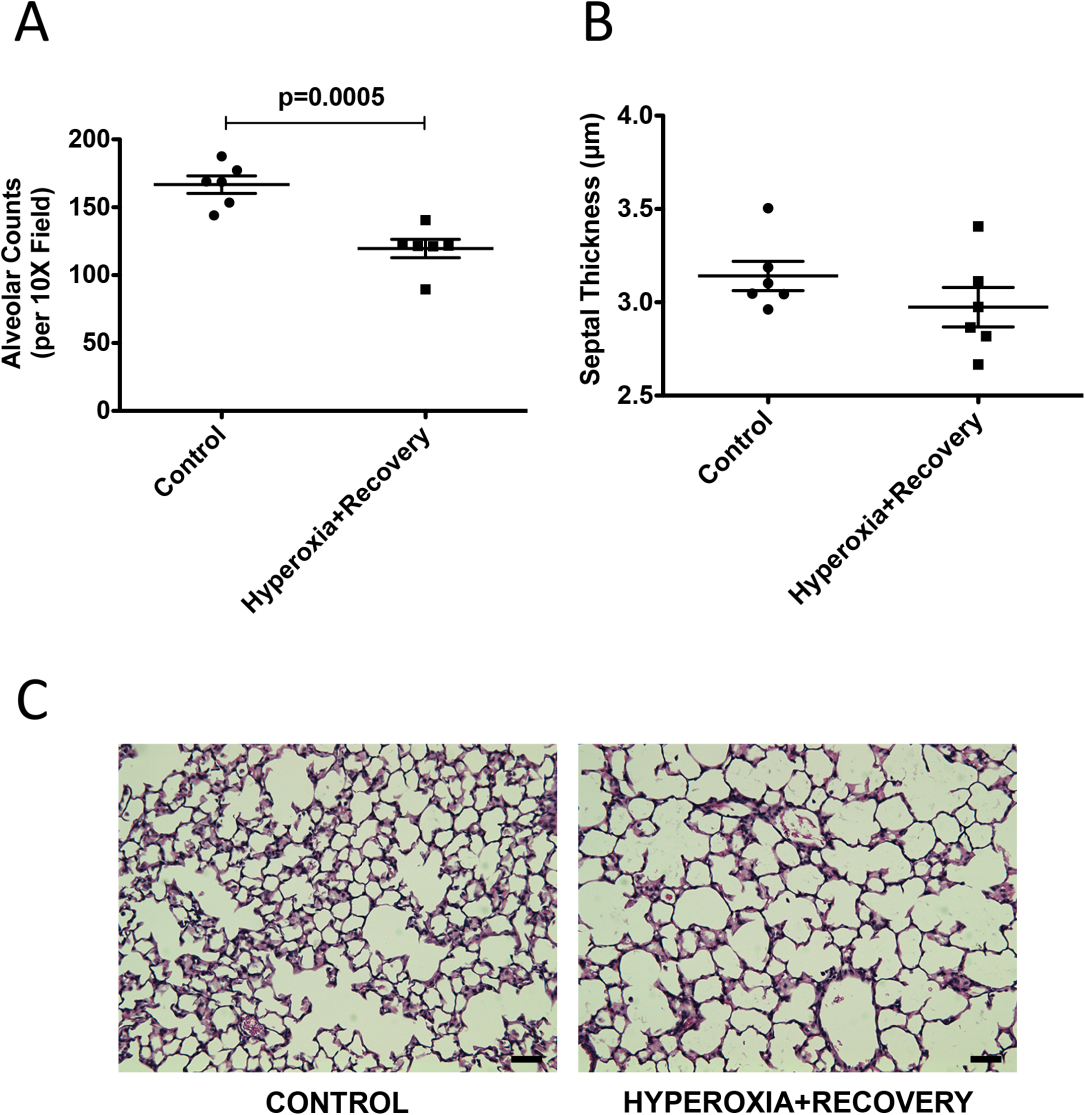
Neonatal hyperoxia exposure is associated with lower alveolar counts in 21d mice. Mice were exposed to hyperoxia or room air for 14d, followed by recovery in room air for an additional 7d (21d). (A) Alveolar counts and (B) septal thickness were measured using research-based digital image analysis software (n = 6 animals per group). (C) Representative H&E lung sections are shown for control 21d and hyperoxia + recovery 21d. Bar = 50 μm. Data are expressed as dot plots with each dot representing individual animals with means and SEM represented by horizontal lines. Results were analyzed using an unpaired t-test.

**Fig 3:**
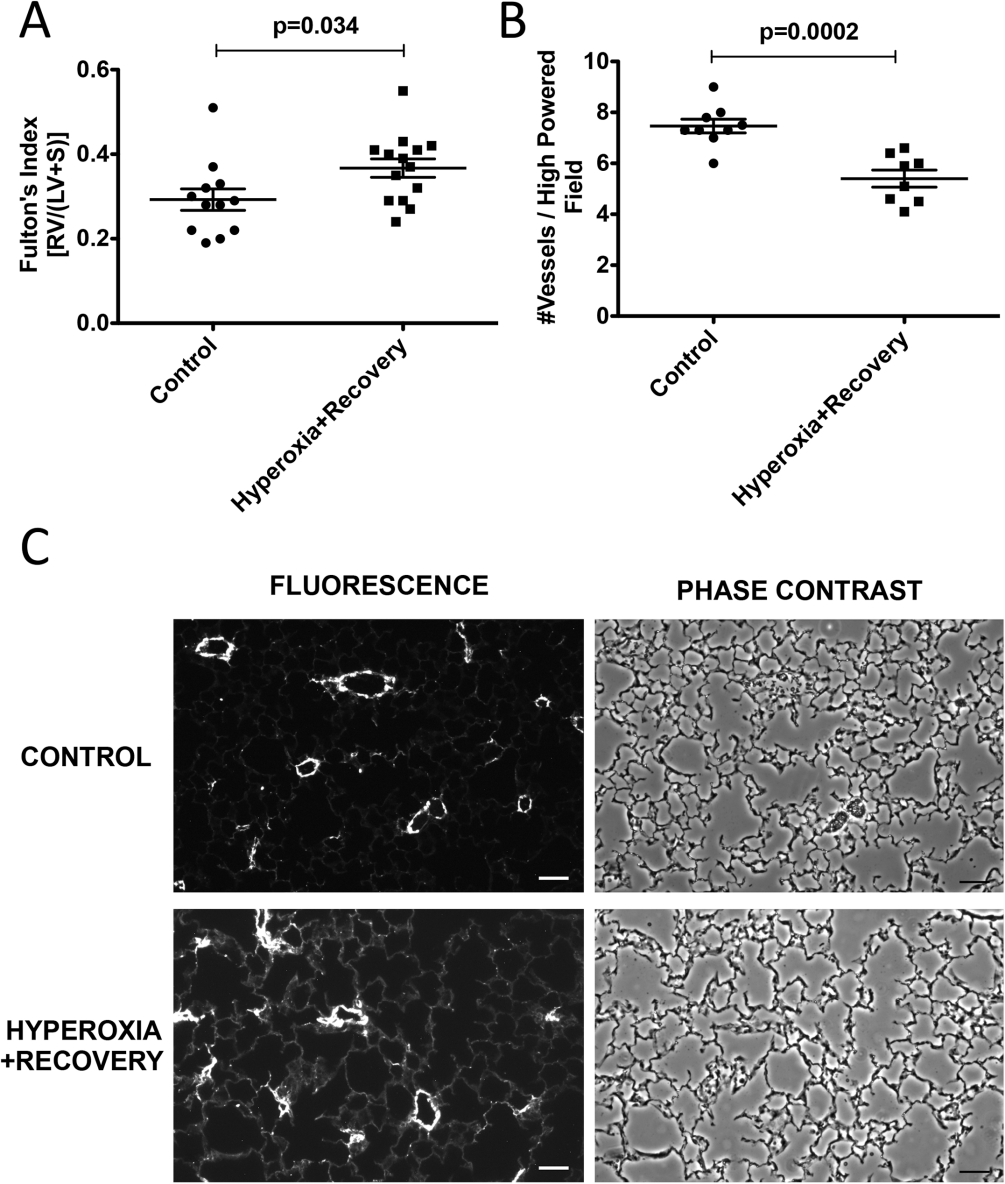
Mice exposed to neonatal hyperoxia have persistent RVH and decreased vessel density at 21d. Mice were exposed to hyperoxia or room air for 14d, followed by recovery in room air for an additional 7d (21d). (A) Fulton’s index (RV weight/LV+S weight) was used to assess RVH (n = 12–14 animals per group). (B) vWF staining was performed on lung sections to evaluate vessel number. Small non-muscularized PAs (<100 μM) were counted and averaged per animal (n = 8–9 animals per group). (C) Representative phase and fluorescent images are shown for 21d control and 21d hyperoxia + recovery. Bar = 50 μm. Data are expressed as dot plots with each dot representing individual animals with means and SEM represented by horizontal lines. Results were analyzed using an unpaired t-test.

Newborn mice were exposed to room air or 75% O_2_ (chronic hyperoxia, CH) for 14d and returned to room air until 21d, when heart and lung tissue were harvested. Alveolar simplification, RVH and decreased vessel density were evident at 14d, and this lung and cardiovascular damage persisted at 21d (Figs 1–3). The mean alveolar area was increased by 23% (529±11 μm^2^ vs. 427±4 μm^2^) (Fig 1A), chord length was increased by 17% (41±1.0 μm vs. 35±0.9 μm) (Fig 1B), and alveolar counts were decreased by 28% (120±7 vs. 167±6) (Fig 2A) as compared to age-matched controls. There were no statistical differences in septal thickness between controls and CH animals (Fig 2B). The representative micrographs of the lung structure are shown in Figs 1C and 2C. The Fulton’s Index was 28% higher (0.37±0.02 vs. 0.29±0.03) when compared to controls (Fig 3A). Histological sections of 21d mouse lungs were stained with vWF. CH mice showed a significant decrease of 27% (5.41±0.33 vessels/high power field (HPF) vs. 7.44±0.27 vessels/HPF) in vessel density compared to age-matched controls (Fig 3B). Representative micrographs of the vessel counts are shown in Fig 3C.

### CH mice have vessel remodeling that persists at 21d

**Fig 4:**
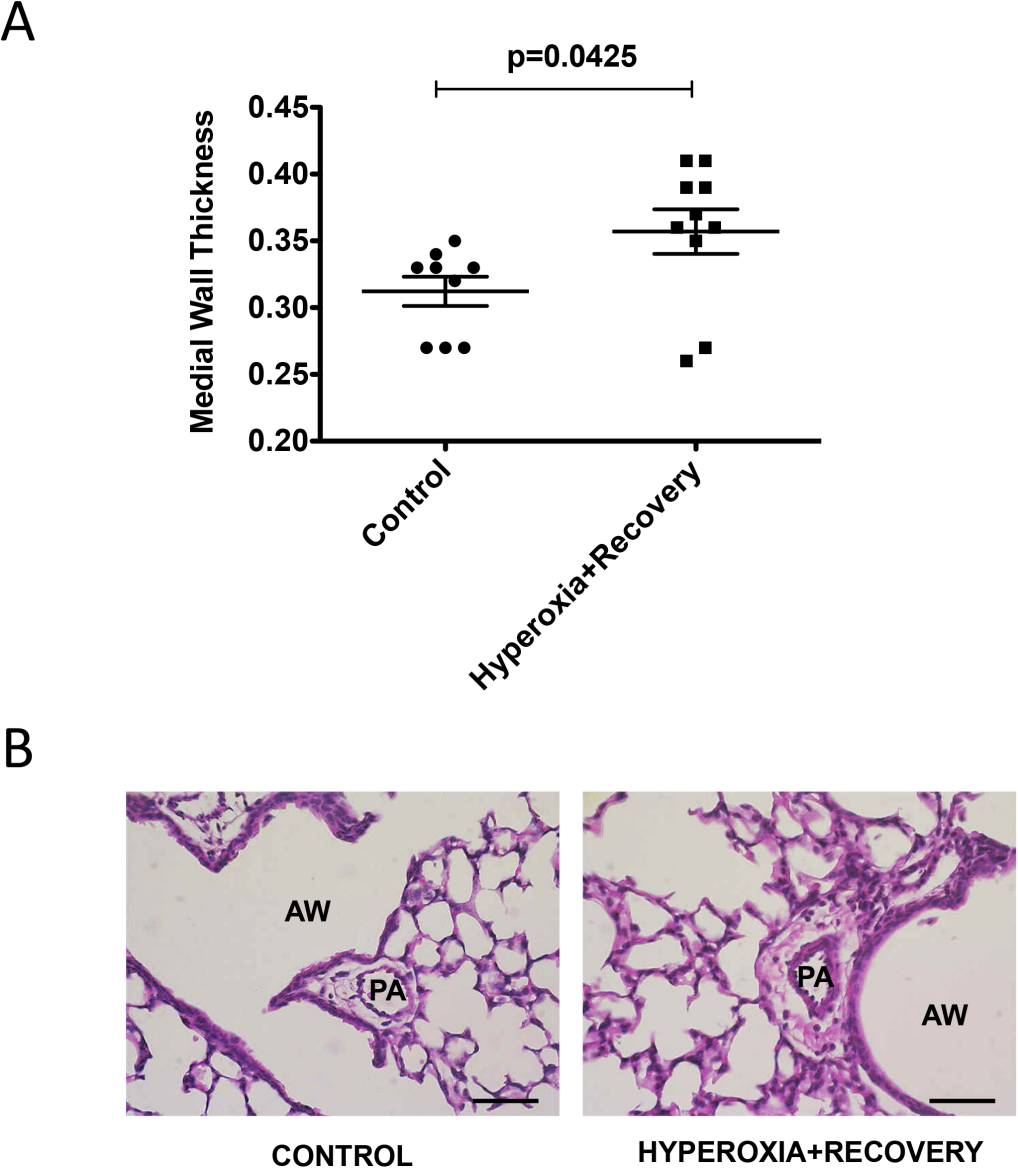
Mice exposed to hyperoxia during the neonatal period have persistently elevated medial wall thickness at 21d. Mice were exposed to hyperoxia or room air for 14d, followed by recovery in room air for an additional 7d (21d). (A) MWT was measured as the ratio of the small PA wall area over total PA area (n = 9–10 animals per group). (B) Representative H&E-stained lung sections are shown for control 21d and hyperoxia + recovery 21d. Bar = 50 μm. Data are expressed as dot plots with each dot representing individual animals with means and SEM represented by horizontal lines. Results were analyzed using an unpaired t-test. PA = Pulmonary Artery, AW = Airway.

Lungs from CH mice and aged-matched controls were inflation fixed and stained to assess medial wall thickness as an indirect measure of pulmonary hypertension. 21d CH mice still had subtle but significant evidence of vessel remodeling: medial wall thickness (MWT) was increased by 16% (0.36±0.02 vs. 0.31±0.01) compared to controls (Fig 4A). Representative micrographs of pulmonary arteries are shown in Fig 4B.

### CH mice have disrupted components of cGMP signaling

**Fig 5:**
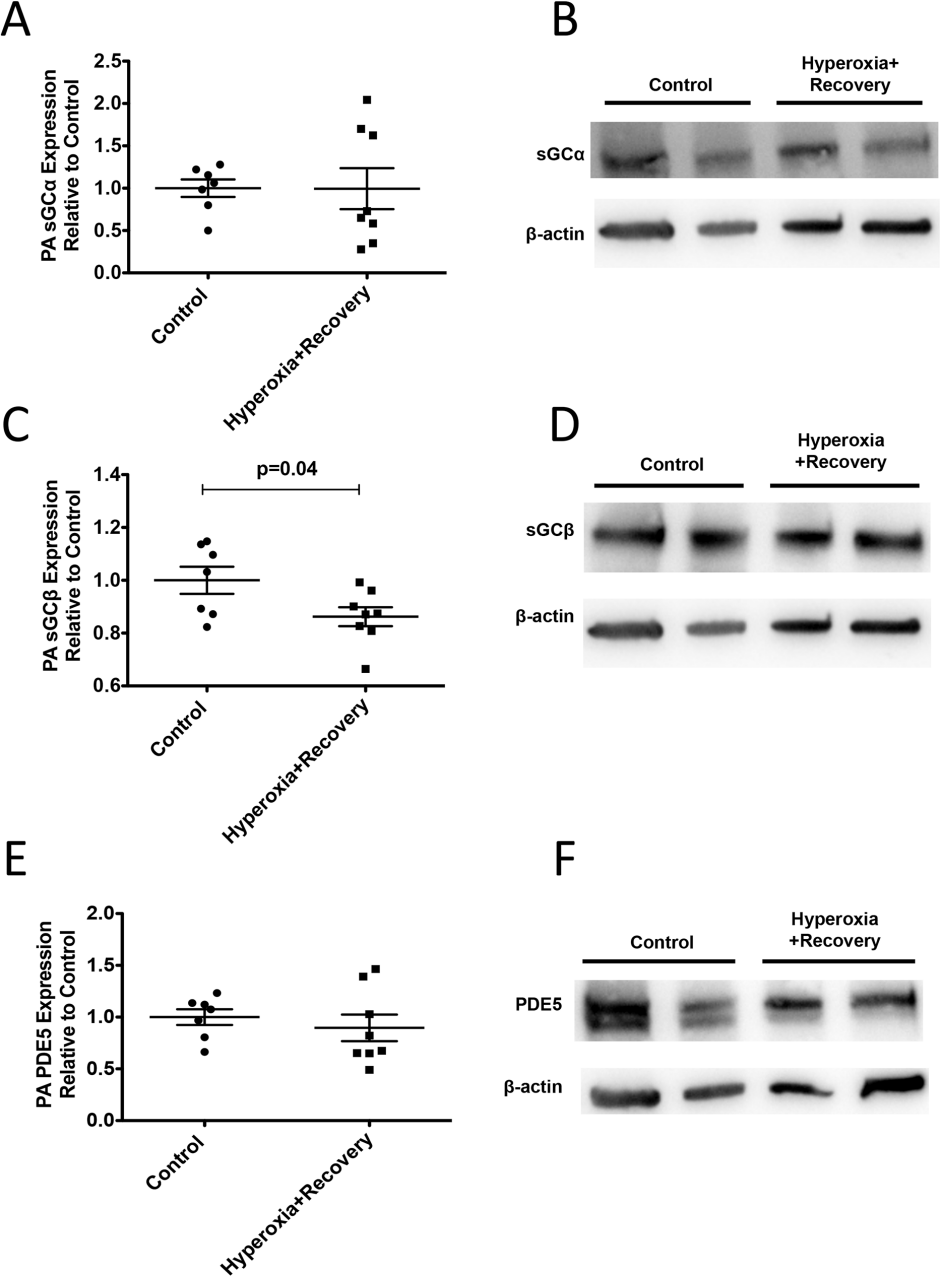
PA sGCβ expression is persistently decreased after neonatal hyperoxia exposure. Mice were exposed to hyperoxia or room air for 14d, followed by recovery in room air for an additional 7d (21d). PA protein expression of sGCα (A), sGCβ (C), and PDE5 (E) were measured by western blot and normalized to β-actin (n = 7–8 animals per group). Representative Western blots are shown for sGCα (B), sGCβ (D), PDE5 (F), and corresponding β-actin. Data are shown as fold changes relative to untreated controls and are expressed as dot plots with each dot representing individual animals with means and SEM represented by horizontal lines. Results were analyzed using an unpaired t-test.

**Fig 6:**
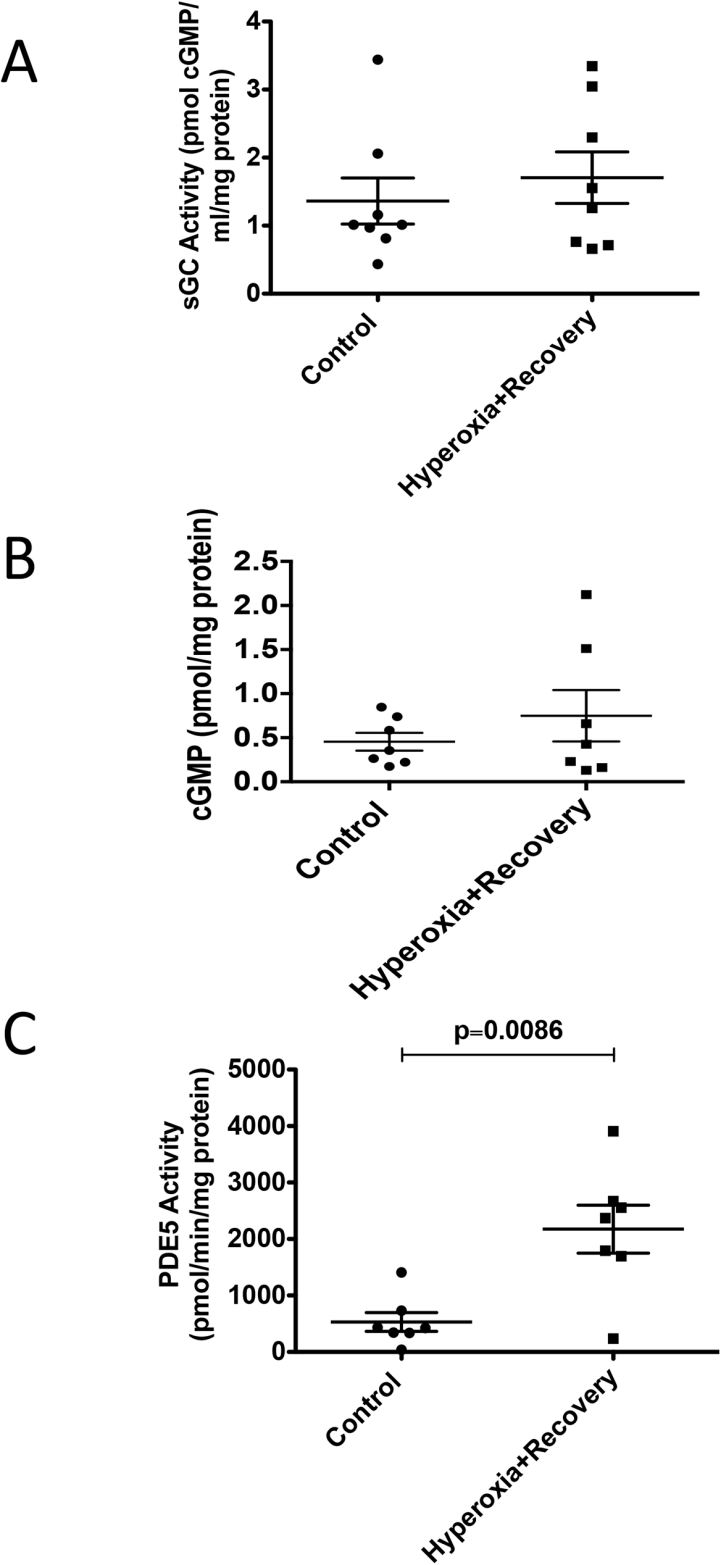
Neonatal hyperoxia exposure is associated with persistent abnormalities in pulmonary artery cGMP signaling. Mice were exposed to hyperoxia or room air for 14d, followed by recovery in room air for an additional 7d (21d). (A) sGC activity was measured as previously described [6] (n = 8 animals per group). (B) PA lysates were assayed for cGMP by enzyme-linked immunoassay, and cGMP was normalized for milligrams of total protein (n = 7 animals per group). (C) PDE5 specific activity was measured as the sildenafil-inhibitable fraction of total cGMP hydrolysis, normalized for total milligrams of protein (n = 7 animals per group). Data are expressed as dot plots with each dot representing individual animals with means and SEM represented by horizontal lines. Results were analyzed using an unpaired t-test.

We isolated PA protein from 21d mice and evaluated the lysates for protein expression of sGCα, sGCβ, and PDE5 (Fig 5) as well as for sGC activity, cGMP levels, and PDE5 activity (Fig 6). The small PAs from 21d CH mice showed a modest decrease in sGCß expression (0.86±0.04 fold control vs. 1±0.05 fold control), but did not show any significant difference in either sGC alpha expression or sGC activity (Figs 5A, 5C, and 6A). However, while PDE5 protein expression was unchanged in CH mice relative to controls (Fig 5E), PDE5 activity was significantly elevated by 4.1 fold (2175±424 pmol cGMP hydrolyzed/min/mg protein vs. 531±165 pmol cGMP hydrolyzed/min/mg protein) in CH mice compared to controls at 21d (Fig 6C). Finally, total cGMP levels in the isolated small PAs were not significantly different than room air controls (Fig 6B).

### Sildenafil administered during room-air recovery does not improve alveolar simplification, decreased vessel density or vessel remodeling caused by chronic neonatal oxygen exposure

**Fig 7:**
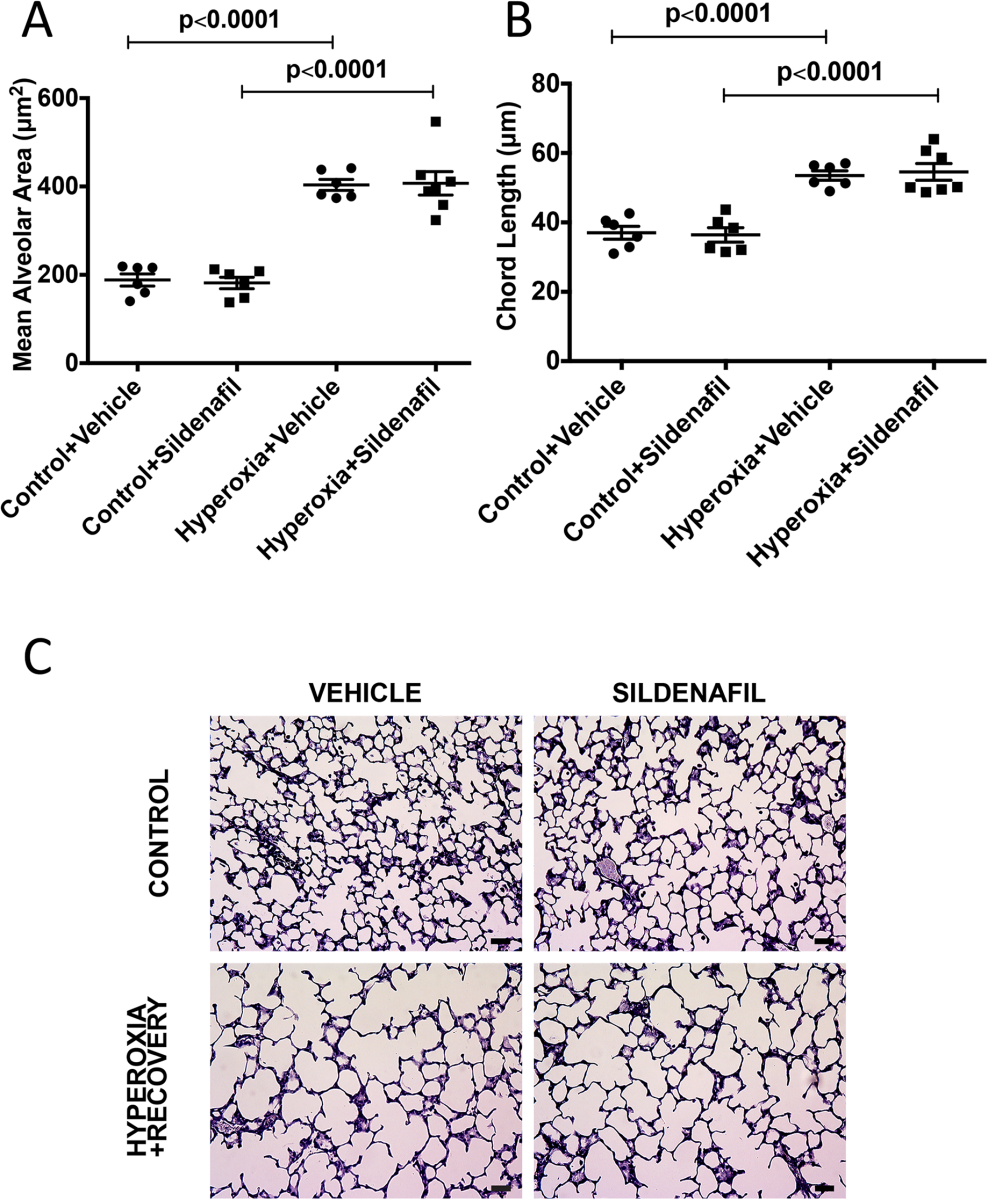
Sildenafil treatment during recovery does not ameliorate alveolar simplification. Mice were exposed to hyperoxia or room air for 14d, followed by recovery in room air for an additional 7d ± sildenafil or vehicle. Hematoxylin-stained sections were imaged, and (A) mean alveolar area and (B) chord length were measured (n = 6–7 animals per group). (C) Representative hematoxylin-stained lung sections are shown for vehicle-treated control, vehicle-treated hyperoxia + recovery, sildenafil-treated control, and sildenafil-treated hyperoxia + recovery. Bar = 50 μm. Data are expressed as dot plots with each dot representing individual animals with means and SEM represented by horizontal lines. Results were analyzed by ANOVA with post-hoc Bonferroni’s analysis.

**Fig 8:**
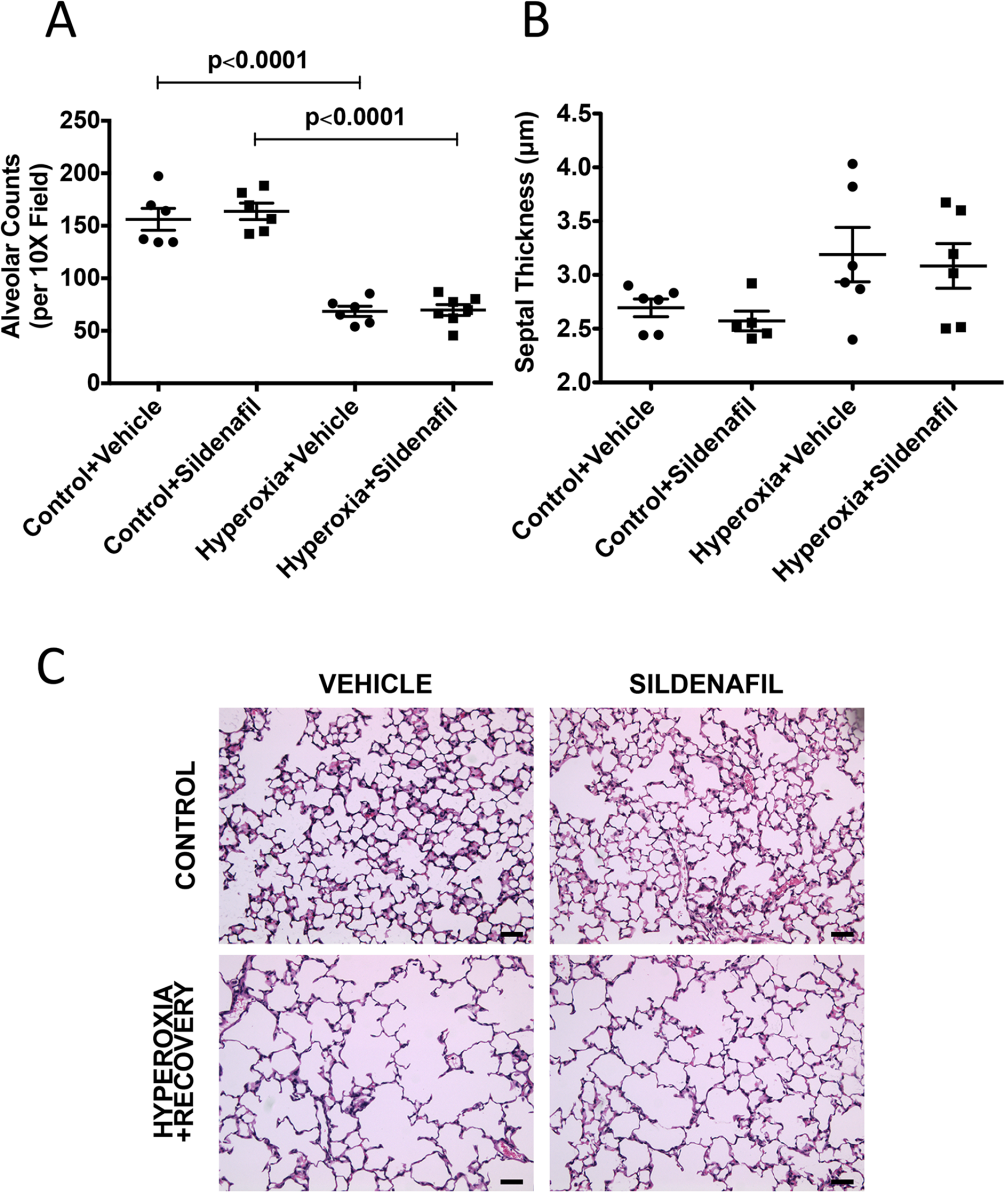
Sildenafil treatment during recovery does not affect hyperoxia-induced decreases in alveolar counts. Mice were exposed to hyperoxia or room air for 14d, followed by recovery in room air for an additional 7d ± sildenafil or vehicle. H&E sections were imaged, and (A) alveolar counts and (B) septal thickness were measured (n = 5–7 animals per group). (C) Representative H&E lung sections are shown for vehicle-treated control, vehicle-treated hyperoxia + recovery, sildenafil-treated control, and sildenafil-treated hyperoxia + recovery. Bar = 50 μm. Data are expressed as dot plots with each dot representing individual animals with means and SEM represented by horizontal lines. Results were analyzed by ANOVA with post-hoc Bonferroni’s analysis.

**Fig 9:**
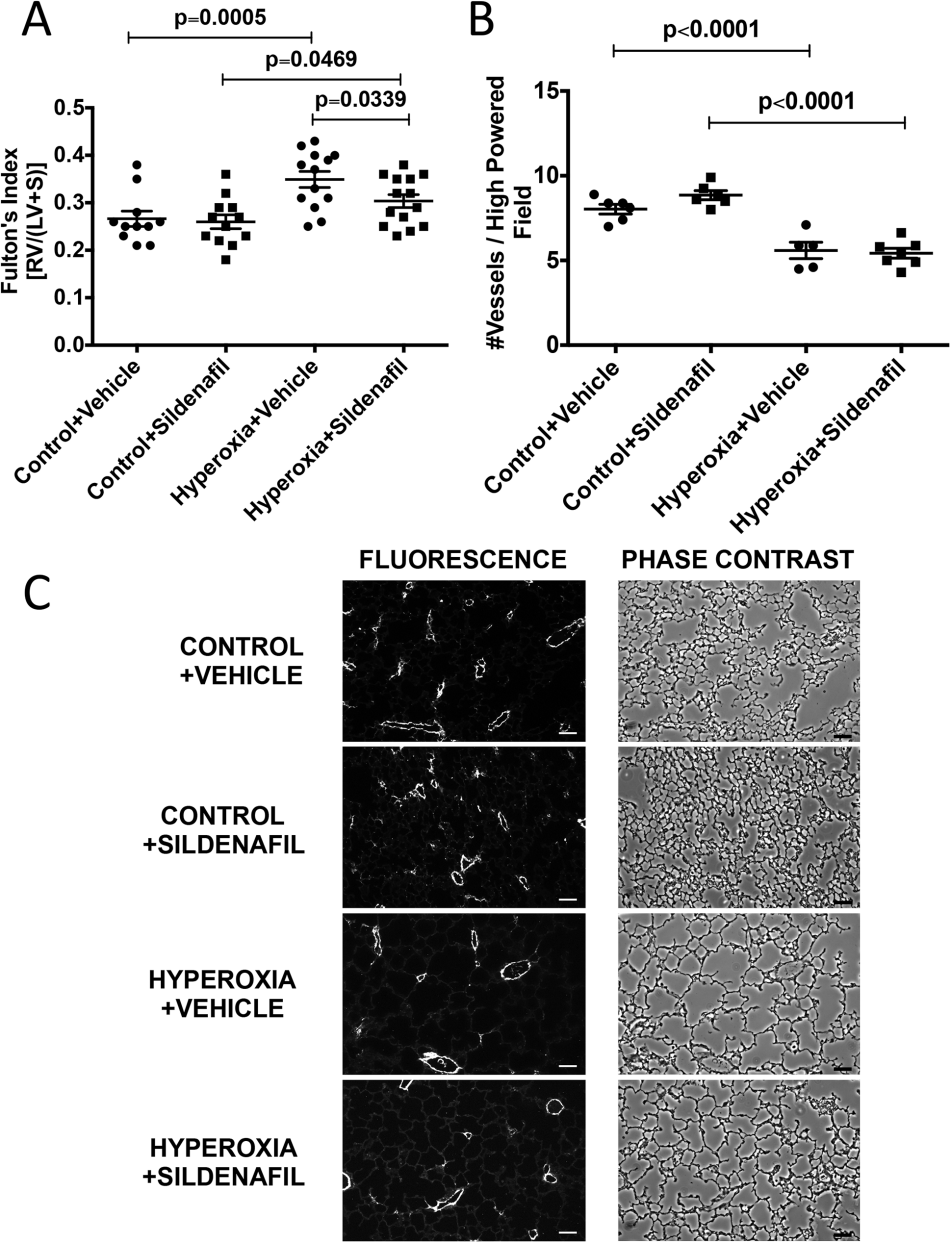
Sildenafil administered during room air recovery improves hyperoxia-induced RVH but does not impact vessel counts. Mice were exposed to hyperoxia or room air for 14d, followed by recovery in room air for an additional 7d ± sildenafil or vehicle. (A) Fulton’s index (RV weight/LV+S weight) was used to assess RVH (n = 11–14 animals per group). (B) vWF staining was performed on lung sections to evaluate vessel number. Small non-muscularized PAs (<100 μM) were counted and averaged per animal (n = 5–7 animals per group). (C) Representative phase and fluorescent images are shown for vehicle-treated control, vehicle-treated hyperoxia + recovery, sildenafil-treated control, and sildenafil-treated hyperoxia + recovery. Bar = 50 μm. Data are expressed as dot plots with each dot representing individual animals with means and SEM represented by horizontal lines. Results were analyzed by ANOVA with post-hoc Bonferroni’s analysis.

**Fig 10:**
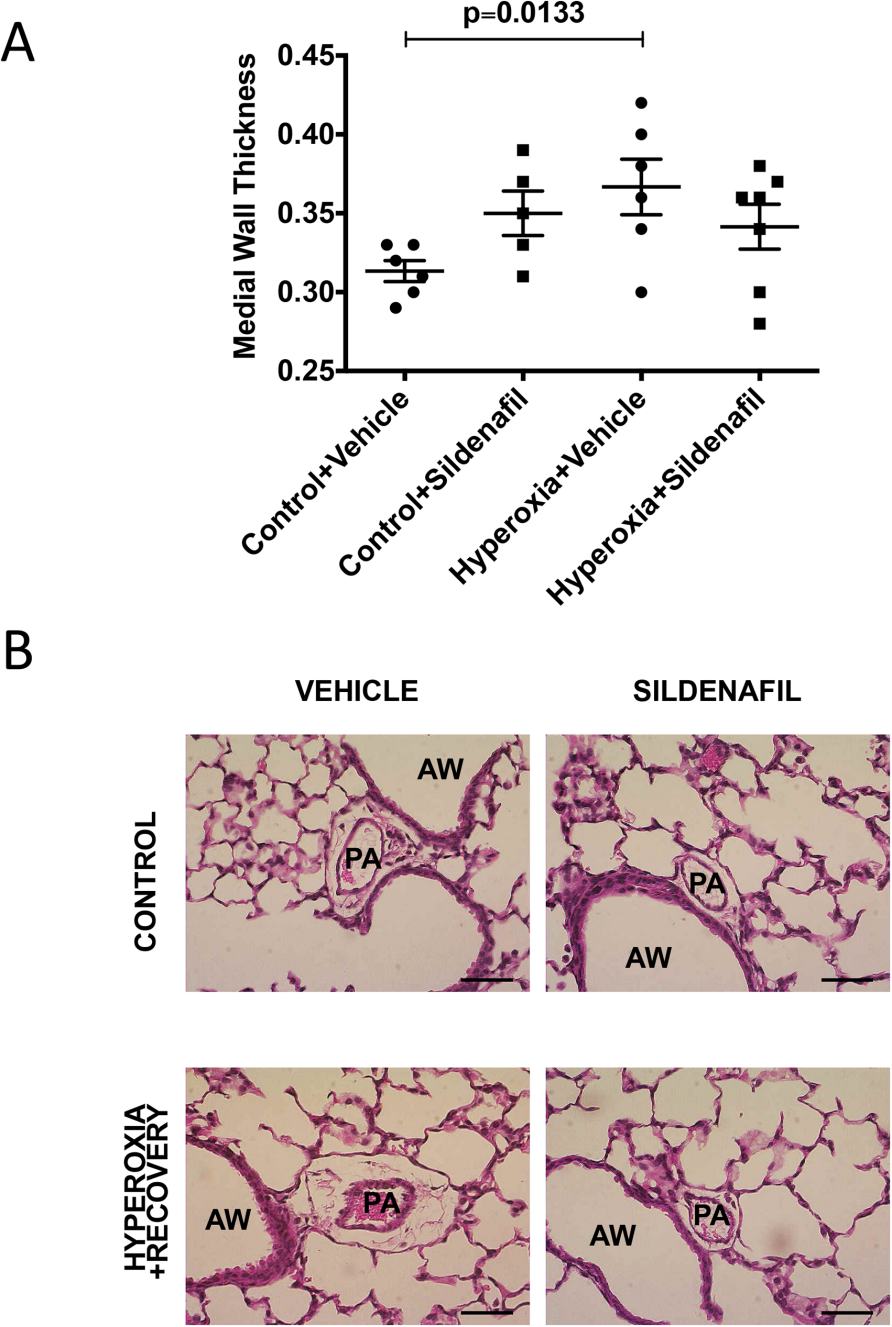
Sildenafil treatment during recovery does not ameliorate increased MWT associated with neonatal hyperoxia exposure. Mice were exposed to hyperoxia or room air for 14d, followed by recovery in room air for an additional 7d ± sildenafil or vehicle. (A) H&E stained sections were imaged, and MWT was measured as the ratio of the small PA wall area over total PA area (n = 6–7 animals per group). (B) Representative H&E-stained lung sections are shown for vehicle-treated control, vehicle-treated hyperoxia + recovery, sildenafil-treated control, and sildenafil-treated hyperoxia + recovery. Bar = 50 μm. Data are expressed as dot plots with each dot representing individual animals with means and SEM represented by horizontal lines. Results were analyzed by ANOVA with post-hoc Bonferroni’s analysis. PA = Pulmonary Artery, AW = Airway.

We have established that 14d of hyperoxia starting at birth causes alveolar simplification, RVH, decreased vessel density, and vessel remodeling that does not resolve with 7d of room air recovery. We subsequently treated these mice with subcutaneous sildenafil injections during the room-air recovery period. Mice that were treated with sildenafil did not show any significant improvement in their alveolar structure, vessel density or vessel remodeling when compared to vehicle-treated control animals (Figs 7–10). Representative micrographs of lung architecture, vessel counts, and pulmonary arteries are shown in Figs 7C, 8C, 9C and 10B, respectively.

### Sildenafil administered during room-air recovery improves RVH

21d CH mice had significant RVH compared to room-air controls (0.35±0.02 vs. 0.27±0.02) (Fig 9A). Sildenafil-treated CH mice showed a significantly improved Fulton’s Index compared to CH+vehicle mice (0.30±0.01 vs. 0.35±0.02) (Fig 9A). Thus, sildenafil administered during the room-air recovery period significantly attenuates RVH, but does not repair other components of the lung and cardiovascular damage caused by neonatal hyperoxia exposure.

### Sildenafil attenuates PDE5 activity in the small PAs of CH mice

**Fig 11:**
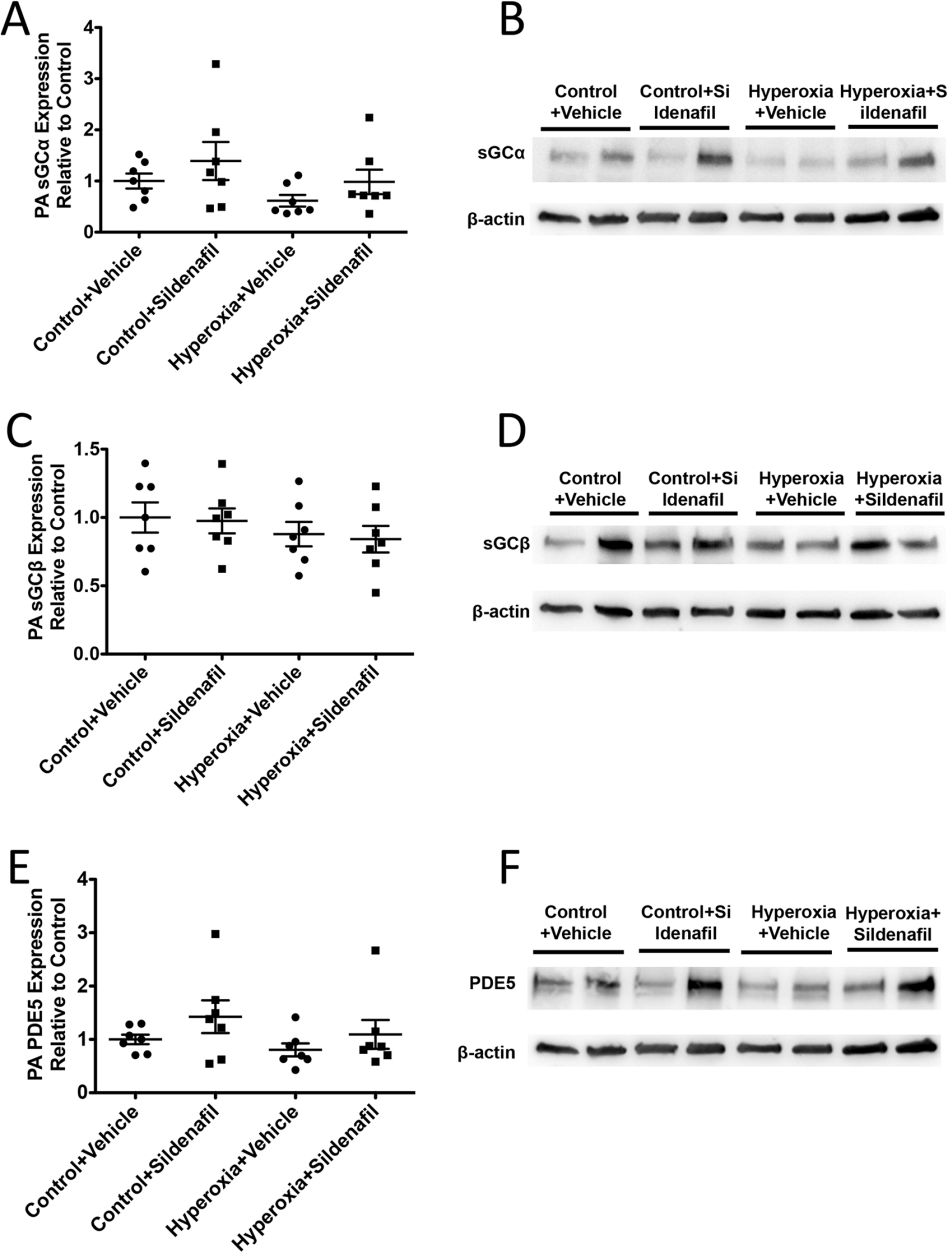
Sildenafil treatment during room air recovery does not affect PA protein expression of sGα, sGCβ, or PDE5. PA protein expression of sGCα (A), sGCβ (C), and PDE5 (E) were measured by western blot and normalized to β-actin (n = 7 animals per group). Representative Western blots are shown for sGCα (B), sGCβ (D), PDE5 (F), and corresponding β-actin. Data are shown as fold changes relative to vehicle-treated controls and are expressed as dot plots with each dot representing individual animals with means and SEM represented by horizontal lines. Results were analyzed by ANOVA with post-hoc Bonferroni’s analysis.

**Fig 12:**
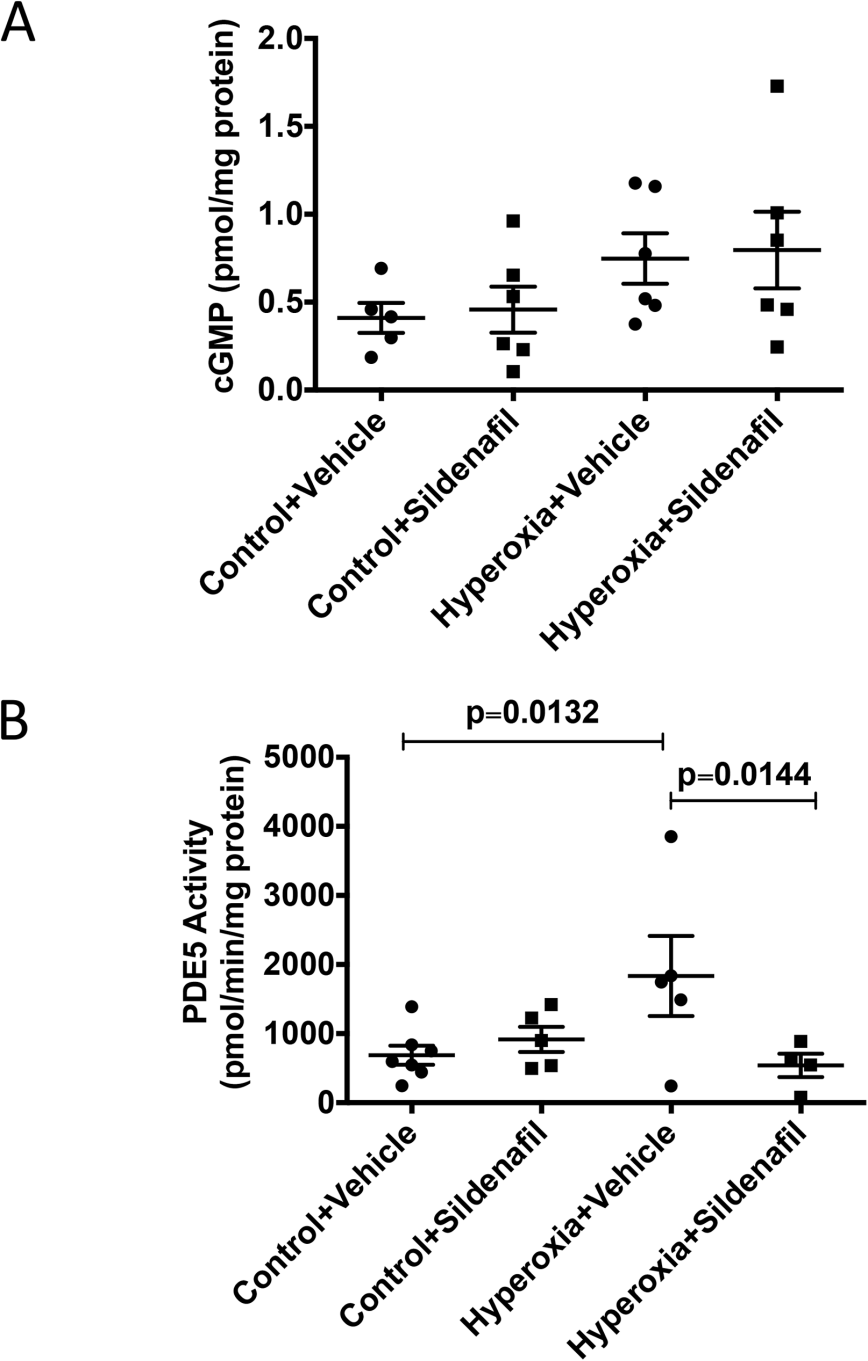
Sildenafil treatment of hyperoxia-exposed mice during recovery attenuates PDE5 activity without significant effects on cGMP levels. Mice were exposed to hyperoxia or room air for 14d, followed by recovery in room air for an additional 7d ± sildenafil or vehicle. (A) PA lysates were assayed for cGMP by enzyme-linked immunoassay, and cGMP was normalized for milligrams of total protein (n = 5–6 animals per group). (B) PDE5 specific activity was measured as the sildenafil-inhibitable fraction of total cGMP hydrolysis, normalized for total milligrams of protein (n = 4–7 animals per group). Data are expressed as dot plots with each dot representing individual animals with means and SEM represented by horizontal lines. Results were analyzed by ANOVA with post-hoc Bonferroni’s analysis.

To evaluate if sildenafil repairs NO-cGMP signaling in the small PAs of 21d mice, we isolated PA protein from mice that were administered sildenafil during the room air recovery period. PA lysates were analyzed for protein expression of sGCα, sGCβ, and PDE5 as well as for cGMP content and PDE5 activity. There were no significant differences in protein expression of sGCα, sGCβ, or PDE5 in either vehicle-treated or sildenafil-treated mice (Fig 11). There was a trend towards increased cGMP levels with sildenafil treatment in both control and hyperoxia groups that did not reach statistical significance (Fig 12A). Hyperoxia-induced PDE5 activity was significantly lower in the small PAs of CH+sildenafil animals compared to the CH+vehicle animals (539±169 pmol cGMP hydrolyzed/min/mg protein vs. 1834±580 pmol cGMP hydrolyzed/min/mg protein)(Fig 12B).

## Discussion

Given the paucity of available treatment options, oxygen therapy remains the cornerstone of BPD treatment. Yet, there is increasing evidence from animal models that neonatal hyperoxia exposure disrupts pulmonary alveolar and vascular development resulting in long-term dysfunction [7–9, 24–28]. Neonatal mice exposed to prolonged hyperoxia develop BPD-like lung injury characterized by alveolar simplification, cardiovascular remodeling, and aberrant cGMP signaling [6]. While it is apparent that even short exposures to oxygen during the neonatal period can lead to persistent injury in rodents [6, 9, 10, 29], it is not clear how long such injury lasts and if recovery can be accelerated through the use of targeted pharmacological treatment. In the present study, we wanted to determine whether the cardiopulmonary and signaling anomalies associated with neonatal hyperoxia exposure persist even after a period of room air recovery. We demonstrate that exposure of neonatal mice to high levels of oxygen during the first two weeks of life leads to persistent pulmonary and cardiovascular anomalies as well as disruptions in pulmonary vascular NO-cGMP signaling that are still apparent at one month of life. This is consistent with other recent studies suggesting that hyperoxia-mediated lung and cardiovascular injury can last up to 8 weeks of age in mice [8, 26–28]. However, to date, no one has investigated potential therapeutic options in established disease, which is where data is desperately needed clinically. In the present study, we show that treatment with low dose sildenafil, a PDE5 inhibitor, during the first week of room air recovery, is associated with more rapid improvement in RVH and decreases in vascular PDE5 activity. However, sildenafil does not improve the rate of lung repair and recovery.

One of the consequences of early life hyperoxic exposure is impairment in alveolar growth, a finding that has been consistently reported in infants with BPD [30]. Similarly, exposure of newborn rodents to high levels of oxygen results in an arrest of alveolar development, leading to alveolar simplification [7, 31]. In agreement with other published reports demonstrating the persistence of alveolar simplification in adult rats and mice exposed to hyperoxia during the neonatal period [8, 26–29, 32], we demonstrate that this impairment in alveolarization persists in our model, showing no significant recovery after a 1 week period of room air recovery (Figs 1 and 2). One group has demonstrated in mice that septal thickness is unchanged after 14d of exposure to 85% O_2_, but is modestly increased after 14d of room air recovery [21], whereas another group using >90% O_2_, showed an increase in septal thickness after 10d of exposure [33]. In rats, some groups have seen increased septal thickness with 100% O_2_, but others have not with 60% O_2_ [34, 35]. Thus, there is some variability in hyperoxia-induced changes in septal thickness that seems dose dependent. In addition, differences in tissue embedding methods have been reported to affect the final analysis of lung structure [36, 37]. In this paper, we did not see the increase in septal thickness after recovery (Fig 2B), likely because we used a more mild oxidative insult with 75% O_2_. In addition to aberrant alveolar growth, BPD is characterized by abnormal cardiovascular and microvascular development [38] but it is unclear how long these vascular changes persist. Similar to infants with BPD who develop pulmonary hypertension, neonatal mice exposed to 2 weeks of hyperoxia demonstrate RVH, thickening of resistance PAs, and decreased lung vascularization [6, 39]. Here we demonstrate that both RVH and vessel numbers continue to be significantly affected at 21d such that the right ventricle remains hypertrophied, and there are fewer small vessels in the lungs (Fig 3). We have recently published that the RVH is not completely resolved until 8 weeks of age in mice exposed to hyperoxia during the neonatal period [26]. Medial wall thickness, a marker of pulmonary vascular remodeling, remains elevated in 21d mice (Fig 4). Another very recent study demonstrated that neonatal rats recovered from a milder hyperoxic insult (60% O_2_) by P28 [34]. Taken together, these results suggest that the timeline of recovery from hyperoxic-cardiopulmonary injury depends both on severity of exposure and genetic background, which is consistent with the variability seen in human infants.

Next, we wanted to determine the mechanism responsible for the persistent cardiovascular and pulmonary vascular changes we observed. Work in animal models of pulmonary hypertension has demonstrated that impairments in the nitric oxide-cGMP pathway play an important role in the development of pulmonary hypertension [6, 40, 41]. Our lab has previously shown that mice exposed to 2 weeks of neonatal hyperoxia demonstrate decreased sGCβ protein expression, decreased sGC activity, increased PDE5 activity, and decreased cGMP levels [6]. In the current study, we found that there is still a striking increase in vascular PDE5 activity evident on day 21 (Fig 6). A very modest decrease in sGCβ protein expression is still present, but interestingly, there appears to be some compensatory increase in sGC activity and cGMP on day 21, suggesting that the mouse vasculature attempts to repair the hyperoxia-mediated damage (Fig 6).

Given the aberrant PDE5 activation, we sought to determine whether treatment with sildenafil, a PDE5 inhibitor, during room air recovery, is sufficient to speed the recovery of any of the persistent pulmonary vascular abnormalities we have observed in our model. PDE5 inhibitors are widely available in the clinical setting and have been utilized as therapy in BPD-associated PH [16–19, 42]. Work from animal models has demonstrated that treatment with high dose sildenafil during hyperoxia exposure promotes alveolarization and attenuates PH in neonatal rat models of hyperoxic lung injury [11–13] and in bleomycin-induced fibrosis mouse model [43]. Sildenafil has also been found to attenuate PH in monocrotaline-induced rat models of PH [14, 15]. However, sildenafil now comes with a Food and Drug Administration (FDA) warning against its use in pediatric patients as a result of clinical studies showing increased mortality in patients over one year of age and with weights > 8 kg taking high doses [44]. Of note, several clinical trials are ongoing currently to address the pharmacokinetics and safety of sildenafil in populations of premature infants (NCT01670136, NCT02421068, and NCT02244528). In light of this clinical landscape, we wanted to determine the effects of low dose sildenafil treatment on established disease in our model. Previous work from our lab has demonstrated that low-dose sildenafil treatment during the period of hyperoxia exposure improves pulmonary vascular remodeling and decreases RVH while also attenuating vascular PDE5 activity and increasing cGMP [6]. Since most infants with BPD do not receive treatment with PDE5 inhibitors until after PH has been established, we wanted to determine if treatment with low dose sildenafil during room air recovery would demonstrate similar benefits. In agreement with our previous findings that neonatal mice given low-dose sildenafil during hyperoxia continue to demonstrate alveolar simplification [6], we did not observe any effects of later administration of low-dose sildenafil on alveolar development (Figs 7 and 8). This is in contrast to other studies in the rat model of hyperoxia-induced lung injury, where much larger doses (100 mg/kg/day) led to improvement of alveolarization [11–13]. Of note, a key difference that might explain this lack of improvement is that we administered sildenafil after hyperoxia exposure, in contrast to the studies investigating high doses in rat models where the drug was administered during oxygen exposure [11]. Also, those studies were done in rats and utilized much higher doses that would far exceed a clinically relevant dose for human infants [45]. Interestingly, there was no beneficial effect of sildenafil on pulmonary vascular remodeling in hyperoxic mice during recovery (Fig 10). This is in contrast with our previous results demonstrating the sildenafil given during hyperoxia can prevent vascular remodeling [6]. Despite no observable benefit of sildenafil on remodeling of small PAs and lack of effects on vessel density, there were clear improvements in hyperoxia-induced RVH (Fig 9A). Combined with recently published studies from our lab demonstrating positive effects of sildenafil on right ventricular PDE5 activity and cGMP levels [26], these findings suggest that sildenafil has direct and distinct effects on the myocardium both during hyperoxia and during room air recovery, independent of its effects on the pulmonary vascular bed.

Finally, we investigated effects of rescue sildenafil on PA protein expression of sGC and PDE5 as well as on pulmonary vascular cGMP concentration and PDE5 activity. Because sildenafil, which impacts downstream PDE5, has not previously been shown to impact upstream sGC activity in cardiovascular tissues or cells [46], we did not investigate effects of sildenafil on sGC activity. Sildenafil treatment did not have any significant effects on protein expression of sGC subunits or PDE5 in the vasculature (Fig 11). It was, however, associated with decreased PDE5 activation and a trend towards increased cGMP levels that did not reach statistical significance (Fig 12). The inability to reach a statistically significant increase in PA cGMP may be due to changes in the activity of other cGMP-hydrolyzing phosphodiesterases present in the pulmonary artery during recovery. Future studies will be needed to investigate this in more detail.

In total, the data in the present study is consistent with clinical data that shows that sildenafil administered to children with established PH is only modestly effective [16–19]. In these studies, sildenafil is generally effective at improving PH by echocardiography, but has little impact on respiratory parameters such as oxygen requirement. This is consistent with our results in this study where sildenafil improved RV recovery, but did not impact alveolarization or vascular development. Our data would suggest that future studies should utilize a multi-drug approach such as combining a PDE5 inhibitor and perhaps an sGC activator such as riociguat to drive increases in cGMP levels. Riociguat has recently been shown to be safe and possibly effective in patients with PH due to congenital heart disease [47]. Alternatively, if other phosphodiesterases are implicated, other phosphodiesterase inhibitors may be required.

In conclusion, we have demonstrated that neonatal hyperoxia exposure leads to persistent alveolar and cardiovascular changes in mice, and that some of these changes can be reversed by treatment with low-dose sildenafil during the period of recovery. Our findings also suggest that in addition to effects on vascular PDE5 activity, sildenafil has distinctive protective effects on right ventricle remodeling. The long-term consequences of early oxygen exposure on the developing lung and myocardium and the best treatment strategies to reverse or lessen those changes deserve future study in order to improve therapeutic options for these high-risk infants.
